# Dental care use in Ontario: the Canadian community health survey (CCHS)

**DOI:** 10.1186/s12903-017-0453-7

**Published:** 2017-12-29

**Authors:** Safoura Zangiabadi, Christy Costanian, Hala Tamim

**Affiliations:** School of Kinesiology and Health Sciences, York University, 4700 Keele Street, Toronto, ON M3J 1P3 Canada

**Keywords:** Oral health, Dental care use, Disparities, Ontario

## Abstract

**Background:**

Oral health is a significant measure of overall health, and regular dental visits are recommended for the maintenance of oral health. The purpose of this study is to determine the pattern (amount and type) of, and factors associated with dental care use among Ontarians.

**Methods:**

Data from the 2014 cycle of the Canadian Community Health Survey was used and analysis was restricted to individuals aged 12 and above residing in Ontario. Dental care use was defined by two distinct outcomes: not visiting a dentist within the past year and visiting a dentist only for emergencies. Multivariable logistic regression was performed to examine the association between socio-demographic, health behavior, oral health, and other health-related factors and the two outcomes.

**Results:**

More than a quarter of participants reported not visiting the dentist in the last year, and 19% reported usually visiting a dentist only for emergencies. Multivariable logistic regression analysis suggested that males, individuals of Aboriginal status, those with low educational attainment, low household income, no dental insurance, who smoked, less frequent teeth brushing, poor health of teeth and mouth, or had diabetes were at a significant increased likelihood of not visiting the dentist within the past year, and only visiting a dentist for emergency care.

**Conclusions:**

Socioeconomic status, self-reported oral health, and general health behaviors were associated with dental care use. These findings highlight the need for focusing efforts toward improving dental care use among Ontarians.

## Background

Oral health is an essential facet of health-related quality of life and a periodic dental visit is an indicator of adequate oral health care utilization [1]. Regular dental visits provide an opportunity to ensure optimal oral health, and to help prevent serious and costly future conditions such as tooth loss, oral cancer and periodontal (gum) disease [2–4]. Moreover, preventive dental behavior is shown to be effective in improving health outcomes among the general population [5], since oral health is also linked to overall health with various studies showing a positive association between poor oral hygiene and pancreatic cancer, respiratory, and cardiovascular diseases [3–6]. The frequency of dental check-ups varies for individuals, depending on their oral health needs, oral health history, and medical status [2]. It is generally recommended to visit the dentist at least once per year [7, 8].

The Canadian dental health care system, unlike the medical care system, is not publicly funded and universal, therefore, dental care expenses are managed through employment based insurance, out-of-pocket payments, or government assistance offering support for low-income groups, children, seniors and welfare recipients [9]. According to the Canadian Health Measures Survey, 62.6% of Canadians have employment-related insurance, 31.9% pay directly out of pocket, and 5.5% are under government public programs [10]. The privatization of dental care not only presents financial barriers in accessing dental services among the lower socioeconomic class, but also among those having a higher economic status, as a consequence of their lack of dental insurance plans [3, 11, 12]. For example, Ramraj et al. [11] found that uninsured middle-income Canadians reported greater financial barriers in accessing dental care compared to their insured counterparts. Furthermore, individuals with lower incomes and without insurance were four times more likely to avoid visiting a dental professional due to financial barriers [13]. Thus, income and dental insurance coverage are known to be the major determinants of dental care use as they mitigate the barriers associated with financial costs [14]. Dental care use, in addition to being influenced by economic factors such as income and insurance coverage, is also related to socio-demographic characteristics. In fact, the Andersen Behavioral Model of Health Care Utilization suggests that age, gender, education, occupation and ethnicity also affect dental care seeking behavior, as well as enabling resources such as income and insurance plans that eliminate financial barriers to dental care use, and need factors such as perceived and actual need for dental care [7].

Conflicting and inconclusive evidence as to the oral health benefits of routinely attending dental care for checkups exists. According to systematic reviews, no high-quality evidence is available to support or refute recommending dental attendance to adults with a frequency of less than once a year or any other specific frequency [15–17] .However, some observational studies have shown that individuals who visit a dentist regularly have better clinical and perceived oral health than their counterparts [18–20]. Therefore, the recommendation to have an oral examination on a regular basis remains a key practice in preventive dentistry.

Despite the importance of regular dental visits in improving oral health and oral health-related quality of life [21], less than two-thirds of Canadians attend routine dental visits each year [1]. In fact, the mean frequency of dental visits varies across Canada with Ontario having the highest mean frequency of dental visits [14].To date, the majority of research has focused on the determinants of dental care use among specific populations in Canada such as, youth, elderly, pregnant women, and those receiving government assistance [7, 22–24]. In fact, there is little to no information available on estimates of dental care use in Ontario, Canada’s most populated province, with approximately 14 million residents, 20% of whom are visible minorities. Therefore, the findings of this study might provide more effective preventive services to improve the oral health of Ontarians. The aim of this study is to examine the factors associated with dental care use in Ontario. Findings of this study might elucidate more effective preventive services to be provided.

## Methods

### Study design and participants

This study utilized data from the 2014 cycle of the Canadian Community Health Survey (CCHS) conducted by Statistics Canada. The CCHS is a cross-sectional survey that aims to provide health-related information at the regional and provincial levels. It contains detailed information on health status, health care utilization, and an overview of health risk factors of the Canadian population. A more detailed description of the CCHS design and sampling procedure can be found on the Statistics Canada Website. The CCHS data collection began in 2000 and was conducted every two years until 2007, when it was operated on an annual basis. The target population of the CCHS consists of individual aged 12 years and older, living in the ten provinces and three territories of Canada. However, people living on Indian reserves, residents of institutions and full-time members of Canadian Forces have been and continue to be excluded as the survey is intended to be representative of individuals residing in private households. The survey had obtained information from 136 health regions covering all of the provinces and territories of Canada. The survey uses the multistage stratified cluster-sampling procedure, and interviews are conducted through telephone, with computer-assisted telephone interview application.

### Data collection

Data collection was made using the CCHS questionnaire designed for computer assisted interviewing (CAI). The survey consisted of a 45-min interview, conducted either over the telephone or in person. Analysis was restricted to CCHS participants from the province of Ontario, as the dental visit and oral health modules were part of the CCHS 2014 cycle’s optional content. Optional content is chosen by provincial and territorial stakeholders in coordination with health regions and is only asked in provinces and territories that selected the module for that year.

### Outcome definition and assessment

Dental care use included two different variables; time of last dental visit, and usual reason for seeing a dentist. Time of last dental visit was measured based on the following question: “When was the last time that you went to a dentist?”. For this question, respondents were provided with options listed as: “less than a year”, “1–2 years”, “2–3 years”, “3–4 years”, “4–5 years”, “more than 5 years” and “never”. The responses were recorded as last dental visit <1 year (regular) or ≥1 year (irregular) [7]. The second outcome, usual reason for visiting a dentist was determined by the question: “Do you usually visit the dentist for check-ups or only emergency care?”. For this question, respondents were provided with the following options: “more than once a year for check-ups”, “about once a year for check-ups”, “less than once a year for check-ups”, and “only for emergency care”, and was categorized as check-ups or emergency [7]. Poor dental care use in this study is defined as irregular dental visits and/or visits only for emergencies, in order to encompass both outcomes when interpreting the results.

### Covariates

The following variables were investigated as possible predictors of dental care utilization: socio-demographic factors (age, sex, marital status, education, aboriginal status, language spoken); socio-economic status (household income, dental insurance, and work status); health behavior factors (smoking status, alcohol use, and teeth brushing frequency); oral health factors (health of teeth and mouth) and other health related factors (perceived general health, diabetes (type 1 or type 2), mood disorders, and stress in life. All variables were self-reported by the individual.

### Statistical analysis

The study sample was limited to individuals aged 12 and above residing in Ontario, and with complete information on optional oral health module that captured information on dental care visit frequency, reason for dental visit and oral health habits. Descriptive statistics of the main outcomes and other parameters were conducted. A chi square test was performed to assess the differences in the proportion of not having dental care within the past year and going for emergency visits only among the different levels of the covariates. Unadjusted odds (ORs) and 95% confidence intervals (CIs) to assess the association between poor dental care use, and socio-demographic, health behavior, oral health related and other indicators were also obtained. Multivariable logistic regression analysis adjusting for all variables in Table 1 was conducted to predict the likelihood of poor dental care use. Adjusted ORs and their 95% CIs were estimated. To account for the complex sampling design, bootstrapping was performed to calculate the 95% CI estimates. Population weights, normalized weights and bootstrap weights were all created by Statistics Canada and provided with the CCHS data file. All analyses were conducted using Stata Data Analysis and Statistical Software (Stata, version 13.0). Statistical significance for all analyses was set at alpha = 0.05 for a two-tailed test.

**Table 1:** Characteristics of the total sample and those with dental visits ≥ 1 year and emergency dental visits in Ontario, the Canadian Community Health Survey (CCHS), 2014

Variables	Total	Dental visit ≥1 year	Unadjusted OR (95% CI)	Emergency visits	Unadjusted OR (95% CI)
	N^a^ (%)	N^b^ (%)		N^b^ (%)	
Socio-Demographic Factors					
Age (years)					
< 18	969,400 (8.3)	94,300 (10.0)	1.00	74,700 (7.9)	1.00
18–34	3,058,900 (26.1)	981,600 (32.6)	4.34 (3.22–5.87)	579,300 (19.4)	2.80 (1.90–4.13)
35–54	3,768,400 (32.2)	951,200 (25.6)	3.08 (2.26–4.20)	587,700 (16.0)	2.21 (1.51–3.23)
≥ 55	3,917,200 33.4)	1,138,800 (30.6)	3.95 (2.95–5.29)	933,800 (26.6)	3.99 (2.77–5.75)
Sex					
Female	5,997,800 (51.2)	1,461,900 (25.0)	1.00	992,000 (17.2)	1.00
Male	5,716,000(48.8)	1,704,000(30.7)	1.33 (1.19–1.48)	1183,400(21.6)	1.33 (1.16–1.53)
Aboriginal Status					
No	10,971,900 (97.4)	2,952,400 (27.6)	1.00	2024,600 (19.2)	1.00
Yes	292,000 (2.6)	99,500 (34.8)	1.40 (1.06–1.83)	78,900 (28.0)	1.64 (1.23–2.18)
Marital Status					
Partner	6,685,900 (57.2)	1,751,300 (26.7)	1.00	1169,500 (18.2)	1.00
No Partner	5,002,400 (42.8)	1,406,400 (29.2)	1.13 (1.01–1.26)	999,500 (20.8)	1.19 (1.04–1.35)
Language					
English and/or French	11,198,100 (98.2)	2,969,800 (27.2)	1.00	2030,100 (18.8)	1.00
Other	210,100 (1.84)	1,131,000 (64.6)	4.88 (2.90–8.23)	98,200 (60.6)	6.64 (3.78–11.7)
Education Level					
Less than high school diploma	2,177,800 (18.9)	709,700 (34.6)	1.00	610,300 (30.3)	1.00
High school diploma/College	2,948,400 (25.6)	904,500 (31.5)	0.87 (0.73–1.04)	615,800 (21.8)	0.64 (0.53–0.77)
University education	6,413,100 (55.6)	1,483,400 (23.5)	0.58 (0.50–0.67)	905,500 (14.4)	0.38 (0.33–0.45)
Household Income					
< $30,000	1,781,000 (15.2)	860,400 (50.5)	1.00	717,800 (43.3)	1.00
$30,000- < $100,000	6,045,800(51.6)	1,770,700(30.2)	0.43 (0.37–0.50)	1,196,300(20.7)	0.34 (0.29–0.40)
$100,000–$150,000 and over	3,886,968 (33.2)	534,900 (14.0)	0.16 (0.14–0.20)	261,400 (6.9)	0.10 (0.08–0.12)
Current Work status					
No	4,587,000 (40.0)	1,364,800 (31.5)	1.00	1096,100 (25.8)	1.00
Yes	6880,1000 (60.0)	1,734,500 (25.4)	0.74 (0.66–0.93)	1043,300 (15.4)	0.53 (0.46–0.60)
Dental Insurance					
No insurance	3,780,800 (34.1)	1,778,800 (47.3)	1.00	1402,800 (38.0)	1.00
Government-sponsored plan	623,600 (5.6)	177,400 (28.7)	0.45 (0.35–0.56)	147,500 (24.0)	0.52 (0.40–0.66)
Employer-sponsored plan	6,119,800 (55.2)	1,010,800 (16.6)	0.22 (0.19–0.25)	544,700 (8.9)	0.16 (0.14–0.19)
Private plan	554,800 (5.0)	82,600 (14.9)	0.19 (0.14–0.27)	46,600 (8.5)	0.15 (0.10–0.23)
Health behavior Factors					
Smoking Status					
No	9,588,700 (82.7)	2,310,600 (24.8)	1.00	1535,100 (16.7)	1.00
Yes	2,011,100 (17.3)	832,200 (42.3)	2.22 (1.94–2.54)	626,300 (32.2)	2.37 (2.05–2.74)
Alcohol Use					
Never	3,032,100 (26.5)	925,100 (32.3)	1.00	713,200 (25.3)	1.00
Less than once a month	2,080,100 (18.2)	662,100 (32.8)	1.02 (0.85–1.23)	478,100 (24.0)	0.93 (0.75–1.15)
At least once per month	2,033,974 (17.8)	486,600 (24.1)	0.67 (0.56–0.80)	313,800 (15.7)	0.55 (0.44–0.68)
At least once per week	4,307,990 (37.6)	1,024,800 (24.1)	0.67 (0.58–0.77)	627,200 (14.9)	0.52 (0.44–0.61)
Teeth brushing frequency					
Twice a day or more	8,822,100 (77.6)	1,981,700 (22.6)	1.00	1264,500 (14.5)	1.00
Once a day or less	2,038,400 (17.9)	740,700 (36.5)	2.00 (1.71–2.28)	580,300 (28.8)	2.40 (2.05–2.80)
Not Applicable(Edentate)	503,700 (4.4)	372,800 (74.6)	10.0 (7.66–13.2)	320,800 (66.7)	11.9 (8.96–15.7)
Oral Health Factors					
Health of teeth and mouth					
Excellent/Very good	6,311,900 (55.1)	1,230,000 (19.6)	1.00	699,700 (11.2)	1.00
Good	3,425,500 (29.9)	1,111,900 (32.7)	2.00 (1.72–2.31)	752,900 (22.4)	2.29 (1.95–2.68)
Fair	1,136,200 (9.9)	487,200 (43.2)	3.12 (2.61–3.73)	404,000 (36.4)	4.53 (3.68–5.58)
Poor	572,200 (5.0)	320,600 (56.9)	5.43 (4.02–7.33)	306,900 (56.9)	10.4 (7.70–14.1)
Other Health related Factors					
Diabetes					
No	10,839,800 (92.6)	2,842,600 (26.9)	1.00	1904,100 (18.2)	1.00
Yes	862,700 (7.4)	321,300 (39.2)	1.75 (1.44–2.13)	269,200 (34.4)	2.36 (1.94–2.87)
Mood disorders					
No	10,705,100 (91.6)	2,826,500 (27.0)	1.00	1922,800 (18.6)	1.00
Yes	985,1000 (8.4)	333,900 (36.0)	1.51 (1.27–1.82)	248,000 (26.9)	1.61 (1.32–1.96)
Perceived General health					
Excellent/Very good	6,930,500 (59.2)	1,561,100 (22.8)	1.00	949,700 (14.0)	1.00
Good	3,311,100 (28.3)	1,032,400 (32.1)	1.60 (1.40–1.81)	747,600 (23.7)	1.91 (1.65–2.21)
Fair	1,040,600 (8.9)	386,500 (39.9)	2.24 (1.84–2.73)	315,600 (33.2)	3.10 (2.52–3.70)
Poor	416,500 (3.6)	181,300 (50.1)	3.39 (2.39–4.81)	159,500 (45.3)	5.09 (3.55–7.29)
Stress in life					
Not at all stressful	1,358,400 (11.6)	375,200 (28.7)	1.00	271,100 (21.0)	1.00
Somewhat stressful	9,874,600 (84.6)	2,627,000 (27.3)	0.93 (0.79–1.09)	1,776,200 (18.7)	0 .86 (0.72–1.03)
Extremely Stressful	444,500 (3.8)	157,600 (37.0)	1.46 (0.979–2.18)	123,000 (29.3)	1.55 (1.02–2.37)

^a^Sample size is estimated using normalized weights; ^b^Frequencies are row percentages estimated using normalized weights

## Results

The analytic sample comprised a total of 20,864 subjects weighted to represent 11,713,800 people, where 27.8% reported not having visited a dentist within the past year, and 19.3% reported usually visiting a dentist only for emergencies (data not shown). Table 1 presents sample characteristics related to patterns of dental care use. Over 50% of participants were female, and 49% were males. More than 30% of participants had no dental insurance. Results of the multivariable logistic regression model are shown in Table 2. Compared to those aged less than 18 years old, older individuals were at increased odds of not having visited a dentist within the past year and having emergency visits. Similarly, males had a 1.43 (95% CI: 1.22–1.67) increased odds of not visiting a dentist within the past year, and usually visiting a dentist for emergency care compared to females (OR = 1.54, 95% CI: 1.22–1.85). Aboriginal people were 1.47 (95% CI: 1.02–2.12) times more likely to visit a dentist for emergency purposes compared to non-Aboriginals. Furthermore, subjects with a private dental insurance plan, a household income of 150,000 CAD and over, a university education, and who consumed alcohol at least once per month, or at least once per week were at significantly reduced odds of having a delayed dental visit and visiting for emergency care purposes. Smokers were 1.40 (95% CI: 1.16–1.66) times more likely to report not visiting a dentist within the last year and visiting for emergency care (OR = 1.49, 95% CI: 1.21–1.85). Moreover, those with less frequent teeth brushing and poor health of teeth and mouth were at increased odds of not visiting a dentist within the last year and visiting for emergency care. Lastly, those with diabetes (either Type 1 or Type 2) were 1.48 (95% CI: 1.11–1.97) times more likely to have emergency dental visits compared to those without diabetes (Table 2).

**Table 2:** Results of the multivariable logistic regression analysis for dental visit ≥1 year and emergency dental visits in the Canadian Community Health Survey (CCHS), 2014

Variables	Dental visit ≥1 year	Emergency visits
	Adjusted OR (95% CI)	Adjusted OR (95% CI)
Socio-Demographic Factors		
Age (years)		
< 18	1.00	1.00
18–34	7.38 (4.59–11.9)	4.83 (2.76–8.44)
35–54	5.24 (3.18–8.63)	4.02 (2.24–7.24)
≥ 55	3.63 (2.26–5.82)	3.77 (2.17–6.55)
Sex		
Female	1.00	1.00
Male	1.43 (1.22–1.67)	1.54 (1.22–1.85)
Language		
English and/or French	1.00	1.00
Other	1.60 (0.84–3.04)	1.40 (0.73–2.67)
Aboriginal Status		
No	1.00	1.00
Yes	1.21 (0.87–1.68)	1.47 (1.02–2.12)
Marital Status		
Partner	1.00	1.00
No Partner	0.89 (0.75–1.05)	0.89 (0.73–1.09)
Education Level		
Less than high school diploma	1.00	1.00
High school diploma/College	0.65 (0.50–0.84)	0.53 (0.40–0.70)
University education	0.62 (0.48–0.78)	0.49 (0.38–0.63)
Household Income		
< $30,000	1.00	1.00
$30,000- < $100,000	0.66 (0.53–0.82)	0.61 (0.49–0.77)
$100,000–$150,000 and over	0.33 (0.25–0.45)	0.27 (0.20–0.36)
Current Work status		
No	1.00	1.00
Yes	1.20 (1.00–1.44)	1.11 (0.89–1.38)
Dental Insurance		
No insurance	1.00	1.00
Government-sponsored plan	0.33 (0.25–0.45)	0.31 (0.22–0.44)
Employer-sponsored plan	0.32 (0.27–0.38)	0.25 (0.21–0.30)
Private plan	0.27 (0.19–0.40)	0.21 (0.13–0.35)
Health behavior Factors		
Smoking Status		
No	1.00	1.00
Yes	1.40 (1.16–1.66)	1.49 (1.21–1.85)
Alcohol Use		
Never	1.00	1.00
Less than once a month	0.90 (0.72–1.13)	0.80 (0.62–1.05)
At least once per month	0.67 (0.53–0.86)	0.58 (0.44–0.77)
At least once per week	0.70 (0.57–0.85)	0.55 (0.43–0.69)
Teeth brushing frequency		
Twice a day or more	1.00	1.00
Once a day or less	1.40 (1.16–1.66)	1.60 (1.30–1.96)
Not Applicable(Edentate)	7.20 (4.93–10.5)	7.15 (4.98–10.3)
Oral Health Factors		
Health of teeth and mouth		
Excellent/Very good	1.00	1.00
Good	1.52 (1.28–1.81)	1.70 (1.38–2.07)
Fair	1.88 (1.48–2.37)	2.58 (1.94–3.43)
Poor	2.53 (1.75–3.66)	4.71 (3.24–6.85)
Other Health related Factors		
Diabetes		
No	1.00	1.00
Yes	1.22 (0.94–1.54)	1.48 (1.11–1.97)
Mood disorders		
No	1.00	1.00
Yes	1.13 (0.87–1.49)	1.05 (0.78–1.42)
Perceived General health		
Excellent/Very good	1.00	1.00
Good	1.03 (0.87–1.23)	1.15 (0 .94–1.41)
Fair	1.09 (0.84–1.41)	1.06(0 .81–1.37)
Poor	1.09 (0.68–1.74)	0 .96 (0.58–1.59)
Stress in life		
Not at all stressful	1.00	1.00
Somewhat stressful	1.02 (0.82–1.27)	1.1 (0.82–1.37)
Extremely Stressful	0.95 (0.51–1.77)	1.1 (0.59–1.98)

## Discussion

This study is the first to examine the characteristics of dental care use in Ontario. The results demonstrated that male gender, Aboriginal status, low educational attainment, low household income, and no dental insurance were significantly positively associated with poor dental care use. Likewise, those who smoked, seldom had alcohol, had less frequent teeth brushing, poor health of teeth and mouth, and had diabetes were at significantly increased odds of poor dental care use.

Among various age groups, those older than 18 years were at increased odds of not visiting a dentist within the past year and visiting only for emergencies. This was in accordance with Bhatti et al. whose results suggested a decline in frequency of dental visit as an individual ages [14]. The findings of this study revealed that females were more likely to visit a dentist within the past year and not for emergency purposes, compared to males. Such gender differences in dental care utilization can be attributed to the positive attitudes and greater knowledge of oral health towards dental visits among females [24]. Also, as suggested by previous studies, females were more proactive in maintaining healthy teeth and gum, and more likely to show preventive dental visit behaviors [24].

Individuals of Aboriginal status were more likely to report visiting a dentist in cases of emergency compared to non-Aboriginal people. This reflects the reluctance of Aboriginals to seek regular dental care and delay receiving immediate treatment until symptoms are severe [25]. This reluctance to use dental care might originate from racialized views and stereotypes toward Aboriginal individuals that make them feel unwelcome at the dental office [25]. The finding of the current study is in concordance with a study by Slater on public oral health care among Aboriginal Australians, that found that those of Aboriginal status were more likely to seek emergency services rather than preventive dental care services [26].

Consistent with previous literature, smokers were found to be less likely to report visiting a dentist within the past year than non-smokers [27]. Our results also suggested that those who smoked usually visited a dentist for emergencies only compared to non-smokers. This could be due in part to the fact that smokers tend to engage in health-seeking behavior rather than preventive care behavior, even though regular dental visits are highly recommended for averting future periodontal disease, tooth loss, and other oral health complexities [28]. Interestingly, our results revealed that those who consumed alcohol at least once per week were less likely to report having poor dental care use. This result was not in concordance with a longitudinal study in Sweden, which found that individuals with high alcohol use reported having more irregular dental visits than those with lower alcohol use In fact, previous studies have been inconsistent on the relationship between alcohol use and oral health, with the literature showing an overall weak association between alcohol consumption and risk of caries and periodontitis [29]. On the other hand, light or moderate alcohol use was found to be positively associated with frequent dental checkups and with having more filled teeth in a study by Wu et al. [30] using NHANES data. Therefore, those who consumed alcohol were more likely to obtain dental fillings. A possible explanation for the relationship between heavy alcohol consumption and dental care use is that alcohol may enhance the release of fluoride from certain restorative materials in fillings, thereby reducing susceptibility to more dental caries by reducing oral cariogenic flora [31], and therefore requiring those who consume it to have less dental visits. Further research is needed to elucidate the role of alcohol consumption duration, patterns and volume in relation to dental care use and oral health outcomes.

The present study identified that less frequent teeth brushing and poor oral health of teeth and mouth were significantly associated with poor dental care use, as expected. This is in concordance with Muirhead et al. [7], who suggested that persons with poor oral health reported less dental care utilization, possibly resulting from the fear of dental cost treatment or the anxiety associated with the pain of treatment. This finding affirms that self-perception of oral health needs rather than actual oral health status is a predictor of dental care use. This fits in with Muirhead et al’s [7] “paradox of need”, where working poor individuals with worse self-rated oral health or who had a perceived need for treatment were poor dental care service users.

We also found that subjects with diagnosed diabetes were more likely to visit the dentist for emergency purposes only. This could be explained by fewer dental visits among diabetic patients due to lack of perceived need, cost barriers, and fear or anxiety [32], which may result in the development of serious dental or periodontal diseases that signifies an urgency for an emergency visit. Moreover, evidence suggests that people with diabetes are at increased prevalence of tooth decay and periodontitis, because of high levels of glucose in saliva that leads to proliferation of bacteria and occurrence of oral health complications [33]. Interventions may need to be tailored to subpopulations within the community who are at high risk of experiencing dental problems.

The study had several strengths. To our knowledge, this is the first provincially representative study that examined the relationship between various demographic, socio-economic, health behavior, oral health and other health related factors and dental care use in Ontario. This study also utilized a large sample size allowing for ample statistical power, with population weights accounting for nonresponse bias. Confounding bias was minimized due to the variety of potential covariates that were controlled for in the analysis. However, some limitations are present. Given that this study is a secondary analysis, no information regarding other important dental health variables such as number of decayed/missing/filled teeth, flossing habits, and depth of periodontal pockets was available. Moreover, all variables, including the main outcome, were self-reported and the accuracy of responses is subject to recall bias. Lastly, the cross-sectional nature of the study design does not allow us to infer causality.

## Conclusions

This study examined the pattern of dental care use and its association with socio-demographic, oral health, and health behavior, and other oral and general health related factors in Ontario. The estimate of not visiting the dentist within the past year was high, at 27.8%, while 19% reported visiting for emergency purposes only. Males, individuals of Aboriginal status, those with low household income, low educational level, no dental insurance, less frequent teeth brushing, poor health of teeth and mouth, smokers, and diabetics were at increased odds of poor dental care use. Further validation work is needed for some of the factors included and not included in this study at the national level. Furthermore, these findings highlight the need for focusing efforts toward improving dental care use among individuals of lower socio-economic background, as well as vulnerable populations such as Aboriginals, and those with chronic diseases such as diabetes in order to achieve better oral health outcomes. Further research can elucidate the effectiveness of such programs in decision making processes associated with dental care utilization among individuals. Also, future interventions are required to change the attitudes and behaviors towards maintaining proper oral health, and implementation of health policy reforms to obtain more affordable dental care.

## Abbreviations

CCHS: Canadian Community Health Survey
CI: Confidence interval
OR: Odds ratio
